# Severe fever with thrombocytopenia syndrome: comparison with scrub typhus and clinical diagnostic prediction

**DOI:** 10.1186/s12879-019-3773-1

**Published:** 2019-02-19

**Authors:** Sang-Won Park, Chang-Seop Lee, Jeong-Han Kim, In-Gyu Bae, Chisook Moon, Yee Gyung Kwak, Baek-Nam Kim, Jae Hoon Lee, Seong Yeol Ryu, Hee-Chang Jang, Jian Hur, Jae-Bum Jun, Younghee Jung, Hyun-Ha Chang, Young Keun Kim, Jeong-Hwan Hwang, Yeon-Sook Kim, Hye Won Jeong, Kyoung-Ho Song, Wan Beom Park, Eu Suk Kim, Myoung-don Oh

**Affiliations:** 1 Department of Internal Medicine, Seoul National University College of Medicine, 103 Daehak-ro, Jongno-gu, Seoul, 03080 the Republic of Korea; 2 Department of Internal Medicine, Boramae Medical Center, Seoul, Republic of Korea; 3 Department of Internal Medicine, Chonbuk National University Medical School, Jeonju, Republic of Korea; 4 Department of Internal Medicine, Gyeongsang National University School of Medicine, Jinju, Republic of Korea; 5 Department of Internal Medicine, Inje University College of Medicine, Busan, Republic of Korea; 6 Department of Internal Medicine, Wonkwang University School of Medicine, Iksan, Republic of Korea; 7 Department of Internal Medicine, Keimyung University Dongsan Medical Center, Daegu, Republic of Korea; 8 Department of Infectious Diseases, Chonnam National University Medical School, Gwangju, Republic of Korea; 9 Department of Internal Medicine, Yeungnam University College of Medicine, Daegu, Republic of Korea; 10 Department of Internal Medicine, Ulsan University Hospital, University of Ulsan College of Medicine, Ulsan, Republic of Korea; 11 Department of Internal Medicine, Hallym University Sacred Heart Hospital, Anyang, Republic of Korea; 12 Department of Internal Medicine, School of Medicine, Kyungpook National University Kyungpook National University Hospital, Daegu, Republic of Korea; 13 Department of Internal Medicine, Yonsei University Wonju College of Medicine, Wonju, Republic of Korea; 14 Department of Internal Medicine, Chungnam National University School of Medicine, Daejeon, Republic of Korea; 15 Department of Internal Medicine, Chungbuk National University College of Medicine, Cheongju, Republic of Korea; 16 Department of Internal Medicine, Seoul National University Bundang Hospital, Seongnam, Republic of Korea

**Keywords:** SFTS, Severe fever with thrombocytopenia syndrome, Scrub typhus, Tsutsugamushi, Korea, Prediction, Score

## Abstract

**Background:**

Severe fever with thrombocytopenia syndrome (SFTS) is emerging in Asian 3 countries, China, Japan and Korea, which are scrub typhus endemic areas, and its incidence is increasing. As the two infections overlap epidemiologically and clinically and the accessibility or sensitivity of diagnostic tests is limited, early clinical prediction may be useful for diagnostic and therapeutic purposes.

**Methods:**

Patients aged ≥16 years who were clinically suspected and laboratory-confirmed to be infected with *Orientia tsutsugamushi* or the SFTS virus in South Korea were enrolled. Clinical and laboratory parameters were compared. Scrub typhus was further subclassified according to the status of eschar and skin rash. An SFTS prediction scoring tool was generated based on a logistic regression analysis of SFTS compared with scrub typhus.

**Results:**

The analysis was performed on 255 patients with scrub typhus and 107 patients with SFTS. At initial presentation, subjective symptoms except for gastrointestinal symptoms, were more prominent in scrub typhus patients. In addition to the characteristic eschar and skin rash, headache was significantly more prominent in scrub typhus, while laboratory abnormalities were more prominent in SFTS. Leukopenia (white blood cell count < 4000/mm^3^; odds ratio [OR] 30.13), thrombocytopenia (platelet count < 80,000 /mm^3^; OR 19.73) and low C-reactive protein (< 1 mg/dL; OR 67.46) were consistent risk factors for SFTS (all *P* < 0.001). A prediction score was generated using these 3 variables, and a score ≥ 2 had a sensitivity of 93.1% (95% confidence interval [CI], 87.9–96.4%) and a specificity of 96.1% (95% CI, 93.8–97.6%) for SFTS.

**Conclusion:**

This prediction scoring tool may be useful for differentiating SFTS from eschar- or skin rash-negative scrub typhus. It is a simple and readily applicable tool with potential for use in primary care settings.

## Background

Severe fever with thrombocytopenia syndrome (SFTS) is an emerging infectious disease that is caused by the SFTS virus (SFTSV); it is endemic in 3 East Asian countries: China, Korea and Japan [1–3]. The incidence of SFTS is increasing, and the case-fatality rate ranges from 5.3 to 32.6% [4–6]; however, there are not yet effective antiviral therapeutics or a vaccine [7]. SFTS was listed as a priority disease that requires urgent research and development by the World Health Organization in 2017 [8]. *Orientia tsutsugamushi* is endemic to these 3 countries, which is a leading cause of treatable non-malarial febrile illness in Asia [9]. In 2017, 10,528 cases of scrub typhus were reported in South Korea. Eschar and a maculopapular skin rash are characteristic findings of this disease and are critical clues for its diagnosis. The case-fatality rate of scrub typhus has a median of 6.0% in untreated cases and 1.4% in treated cases [9].

Although SFTSV and *O. tsutsugamushi* do not share specific vectors, they are transmitted to humans through ticks and mites bites mostly, respectively, during outdoor activities. The ecological differences between vectors may characterize their epidemiological features, including the region of infection and peak epidemic seasons. However, there are considerable overlaps of their epidemiological and clinical features, which makes their differential diagnosis difficult, particularly during the high epidemic season of scrub typhus. Patients with SFTS have the potential to deteriorate during the second week of the illness [5], and early diagnosis of SFTS may lead to early investigational therapeutics and stricter infection control measures to prevent human-to-human transmission [10–13]. However, the sensitivity of diagnostic assays for scrub typhus is low [14]. The confirmatory test for SFTS is usually performed in the national reference laboratory, and a serologic assay for the point of care is not yet commercially available. Therefore, only a high index of clinical suspicion may lead to a rapid clinical decision or an early referral, particularly in primary care settings.

Clinical diagnostic prediction based on the features differentiating SFTS from scrub typhus in endemic areas, particularly during the overlap period, may be clinically useful to guide the diagnostic and therapeutic strategies in the absence of rapid point-of-care diagnostic test. This study compared the clinical and laboratory features of the two diseases and constructed a clinical prediction tool composed of a scoring system for SFTS. We performed several subgroup analyses, including for eschar-negative scrub typhus, which is difficult to suspect clinically because it lacks critical clues.

## Methods

### Patients

Patients in South Korea aged ≥16 years who were clinically suspected and laboratory-confirmed to be infected with *O. tsutsugamushi* or SFTSV were enrolled. Cases of eschar-positive and -negative scrub typhus were prospectively included from 8 community-based hospitals in 2006; part of this study was previously published [15]. Additional patients with only eschar-negative scrub typhus were prospectively included from 6 community-based hospitals from 2009 to 2011; these patients had been thoroughly examined and cared for by the infectious diseases specialists in charge. The participating hospitals in both studies were Chonbuk National University Hospital, Dankook University Hospital, Dongguk University Ilsan Hospital, Ilsan Paik Hospital, Namwon Medical Center, Pusan Paik Hospital, Sanggye Paik Hospital, Sunlin Hospital, Boramae Medical Center, and Wonkwang University Hospital. SFTS cases were retrospectively collected from 36 hospitals nationwide from 2013 to 2015. Part of this study was previously published [5], and part of the hospitals are listed in the Acknowledgements section.

Scrub typhus was confirmed either by eschar- or buffy coat-based polymerase chain reaction (PCR) or by a serologic assay. PCR targeting the variable domains I and II of the 56-kDa antigen gene of *O. tsutsugamushi* was performed using a set of primers (forward: TTT CGA ACG TGT CTT TAA GC; reverse: ACA GAT GCA CTA TTA GGC AA; 1151 bp); the products were sequenced to match the reference genotypes, as described in a previous study [15]. The presence of four-fold or greater changes in the titers of the paired sera from an indirect immunofluorescence antibody assay (IFA) or a passive hemagglutination assay (GreenCross SangA; Yongin city, South Korea) was used as the positive serologic criteria. All sera from patients with confirmed scrub typhus were screened for the co-infection with SFTSV. SFTS is a reportable infectious disease to the Korea Centers for Disease Control and Prevention (KCDC) and all SFTSV infections were confirmed at the KCDC by detecting the M segment gene of the SFTSV RNA using one-step reverse transcription (RT)-PCR as described in a previous study [16].

### Study design

Baseline characteristics and clinical and laboratory parameters were compared between scrub typhus and SFTS to determine the differentiating factors. Scrub typhus was further subclassified into eschar-negative and -positive groups for comparison with SFTS. An SFTS prediction scoring tool was generated based on logistic regression analysis for SFTS. The baseline characteristics included demographic variables, comorbidities, site of infection, season of infection, duration from the onset of illness to first visit, duration of the hospital stay, and in-hospital mortality. The clinical parameters included commonly known symptoms and signs of both diseases such as headache, altered consciousness, cough, dyspnea, gastrointestinal manifestations, skin rash and the presence of a bite wound. The laboratory parameters included a complete blood count and chemistry, which can be easily obtained in primary care settings as a point-of-care testing. The worst values of the clinical and laboratory parameters within 24 h of the initial visit were used. The frequency of major complications during the clinical course was also compared.

Altered mentality was defined as a Glasgow coma scale score < 15. Acute kidney injury was defined as serum creatinine levels ≥2.0 mg/dL and 1.5 times the baseline level [17]. Shock was defined as a mean arterial pressure < 65 mmHg. The categorical cut-offs for the comparison of some laboratory values such as thrombocytopenia (platelet count < 80,000 /mm^3^), aspartate transaminase (AST) ≥400 IU/L and alanine transaminase (ALT) ≥200 IU/L were chosen in view of their mean values in SFTS cases and their kinetics during the clinical course, as shown in a previous study [5]. Geographic location was divided into the western and eastern areas of South Korea. The western area included the Seoul metropolitan area and Gyeonggi, Chungcheong and Cholla provinces, which mostly consist of plain rice fields. The eastern area included Kangwon and Gyeongsang provinces, which mostly consist of hilly and mountainous areas.

### Statistical analysis

Chi-square or Fisher’s exact tests were used to analyze the categorical variables. T-tests or Mann-Whitney U-tests were used to compare the continuous variables. A multivariate logistic regression analysis was performed using the risk factors that were significantly (*P* < 0.05) associated with SFTS or scrub typhus in the univariate analysis and adjusted with the duration from the onset of illness to the initial presentation. The SFTS prediction scoring tool was generated using the logistic regression analysis for estimating odds ratios. The receiver operating characteristic curve was constructed for the scoring model (SPSS v20.0, Armonk, NY: IBM Corp.).

## Results

A total of 362 patients were included in the analysis, including 255 patients with scrub typhus and 107 patients with SFTS. Eschar-positive scrub typhus accounted for 80.4% (205/255) and eschar-negative scrub typhus for 19.6% (50/255) of patients. Scrub typhus was confirmed by PCR in 153 patients and by serology in 102 patients. All patients with scrub typhus (*N* = 255) showed negative results for SFTSV in the sera. Compared to scrub typhus patients, patients with SFTS showed a higher median age (71 years), more comorbidities such as diabetes mellitus and hypertension, a greater tendency toward infection in the summer season, a greater tendency to be infected in the eastern area of South Korea, a shorter duration from the onset of illness to the first visit (median of 4 days), a longer duration of hospital stay (median of 10 days), and a higher case-fatality rate (40.2% vs 0.4%, respectively) (Table 1).

**Table 1:** Baseline characteristics of the subjects (*n* = 362)

Variable	Scrub typhus (*n* = 255)				SFTS	*P* value^c^
	Eschar-positive	*P* value^a^	Eschar-negative	*P* value^b^		
	*n* = 205		*n* = 50		*n* = 107	
Age, years (median, IQR)	60 (49–71)	< 0.001	64.5 (51.2–70.7)	0.004	71 (61–78)	< 0.001
Male gender, n (%)	81 (39.5)	0.044	22 (44.0)	0.387	55 (51.4)	0.054
Comorbidity						
Diabetes mellitus	19 (9.3)	0.027	5 (10.0)	0.200	19/106 (17.9)	0.023
Hypertension	44 (21.5)	0.030	13 (26.0)	0.395	35 (32.7)	0.039
CVA	7 (3.4)	1.000	1 (2.0)	1.000	4 (3.7)	0.771
Congestive heart failure	9 (4.4)	0.342	2 (4.0)	0.593	2 (1.9)	0.254
Chronic liver disease	7 (3.4)	0.272	5 (10.0)	0.013	1 (0.9)	0.119
Asthma/COPD	7 (3.4)	0.583	1 (2.0)	0.665	5 (4.7)	0.474
Solid tumor	6 (2.9)	0.098	2 (4.0)	0.098	0	0.111
None	126 (61.5)	0.121	26 (52.0)	0.969	56 (52.3)	0.202
Seasonal occurrence, n (%)						
Spring-summer (Mar-Aug)	0	< 0.001	0	< 0.001	67 (62.6)	< 0.001
Autumn (Sep-Dec)	205 (100)		50 (100)		40 (37.4)	
Geographical location						
Western area	167 (81.5)	< 0.001	42 (84.0)	< 0.001	36/104 (34.6)	< 0.001
Eastern area	38 (18.5)		8 (16.0)		68/104 (65.4)	
Duration, mean (±SD), days						
From onset of illness to admission	6.63 (3.918)	< 0.001	6.38 (5.103)	0.006	4.39 (3.66))	< 0.001
Hospital stay	6.26 (11.927)	< 0.001	6.18 (3.757)	< 0.001	12.07 (9.57)	< 0.001
Mortality, in-hospital	1 (0.5)	< 0.001	0	< 0.001	43 (40.2)	< 0.001

*Abbreviations*: *SFTS* severe fever with thrombocytopenia syndrome, *IQR* interquartile range, *SD* standard deviation, *CVA* cerebrovascular accident, *COPD* chronic obstructive lung disease

^a^*P* value when compared to SFTS

^b^*P* value when compared to SFTS

^c^*P* value when compared to all scrub typhus

At the initial presentation, the overall subjective symptoms, except for gastrointestinal symptoms, were more prominent in patients with scrub typhus. Skin rash was predominantly present in scrub typhus cases (87.8 and 78.0% in eschar-positive and -negative scrub typhus respectively, vs 5.7% in SFTS). Bite wounds were present in 28.3% of SFTS patients. Altered mentality was more common in SFTS patients (27.9%). In the subgroup comparisons, patients with eschar-negative scrub typhus presented fewer subjective symptoms. Fever was present in all the patients and was the initial chief problem leading to a hospital visit. The presence of skin rash (78.0%) in eschar-negative scrub typhus was similar to the rate in eschar-positive scrub typhus (*P* = 0.110) (Table 2). Laboratory abnormalities were more prominent in SFTS. Leukopenia, thrombocytopenia, and an elevation of AST and lactate dehydrogenase (LDH) were more common in SFTS. C-reactive protein (CRP) was rarely elevated in SFTS (mean 1.24 mg/dL) (Table 3). SFTS was associated with a disproportionately higher incidence of major complications during the hospitalization course such as decreased mentality (59.0%), seizure (16.2%), pneumonia (74.4%), the need for mechanical ventilation (32.1%), and acute kidney injury (21.5%) (Table 4).

**Table 2:** Clinical symptom and sign at the initial presentation (*n* = 362)

Variable	Scrub typhus (*n* = 255)				SFTS	*P* value^c^
	Eschar-positive	*P* value^a^	Eschar-negative	*P* value^b^	*n* = 107	
	*n* = 205		*n* = 50			
Fever	205 (100)	< 0.001	50 (100)	0.017	95/106 (89.6)	< 0.001
Headache	179 (87.3)	< 0.001	30 (60.0)	< 0.001	26/105 (24.8)	< 0.001
Myalgia	172 (83.9)	< 0.001	29 (58.0)	0.794	58/104 (55.8)	< 0.001
Conjunctival injection	78 (38.0)	< 0.001	6 (12.0)	0.215	6/98 (6.1)	< 0.001
Sore throat	74 (36.1)	< 0.001	11 (22.0)	0.005	7/105 (6.7)	< 0.001
Cough	76 (37.1)	< 0.001	15 (30.0)	0.004	12/105 (11.4)	< 0.001
Dyspnea	47 (22.9)	0.004	9 (18.0)	0.138	10/104 (9.6)	0.006
Nausea/vomiting	81 (39.5)	0.463	14 (28.0)	0.370	37/105 (35.2)	0.718
Abdominal pain	58 (28.3)	0.019	9 (18.0)	0.778	17/105 (16.2)	0.033
Arthralgia	62 (30.2)	< 0.001	4 (8.0)	0.429	5/104 (4.8)	< 0.001
Skin rash	180 (87.8)	< 0.001	39 (78.0)	< 0.001	6/105 (5.7)	< 0.001
Presence of bite wound	205 (100)	< 0.001	0 (0)	< 0.001	30/106 (28.3)	< 0.001
Altered mentality	11 (5.4)	< 0.001	5 (10.0)	0.012	29/104 (27.9)	< 0.001
Shock (MAP < 65 mmHg)	15 (7.3)	0.085	4 (8.0)	0.427	14/105 (13.3)	0.079

*Abbreviations*: *SFTS* severe fever with thrombocytopenia syndrome, *MAP* mean arterial pressure

^a^*P* value when compared to SFTS

^b^*P* value when compared to SFTS

^c^*P* value when compared to all scrub typhus

**Table 3:** Laboratory findings at the initial presentation (*n* = 362)

Variable	Scrub typhus (*n* = 255)				SFTS	*P* value^c^
	Eschar-positive	*P* value^a^	Eschar-negative	*P* value^b^	*n* = 107	
	*n* = 205		*n* = 50			
White blood cells, /mm^3^	6909 ± 3755	< 0.001	8426 ± 4207	< 0.001	2317 ± 1930	< 0.001
Leukopenia (< 4000/mm^3^), %	42 (20.5)	< 0.001	5 (10.0)	< 0.001	97/106 (91.5)	< 0.001
Hemoglobin, g/dL	12.8 ± 1.8	0.007	12.1 ± 1.6	< 0.001	13.4 ± 1.7	0.001
Anemia (< 11 g/dL)	22 (10.7)	0.532	11 (22.0)	0.019	9/106 (8.5)	0.230
Platelet (×10^3^/mm^3^)	133 ± 47	< 0.001	161 ± 89	< 0.001	61 ± 30	< 0.001
Thrombocytopenia^d^, %	21 (10.2)	< 0.001	5 (10.0)	< 0.001	83/106 (78.3)	< 0.001
Serum albumin, d/dL	3.5 ± 0.6	0.575	3.07 ± 0.59	0.031	3.4 ± 0.6	0.851
Total bilirubin, mg/dL	0.93 ± 1.14	< 0.001	0.64 ± 0.66	0.034	0.40 ± 0.61	< 0.001
AST, IU/L	108 ± 107	< 0.001	170 ± 277	0.007	381 ± 505	< 0.001
≥ 400 IU/L	4/204 (2.0)	< 0.001	4 (8.0)	0.001	33/106 (31.1)	< 0.001
ALT, IU/L	96 ± 108	0.026	138 ± 192	0.824	131 ± 159	0.135
≥ 200 IU/L	16/204 (7.8)	0.002	9 (18.0)	0.789	21/106 (19.8)	0.010
Lactate dehydrogenase, IU/L	700 ± 423	< 0.001	761 ± 393	0.014	1615 ± 2159	< 0.001
≥ 800 IU/L	48/183 (26.2)	< 0.001	14/40 (35.0)	0.021	55/97 (56.7)	< 0.001
Serum creatinine, mg/dL	1.04 ± 0.63	0.634	1.14 ± 0.70	0.567	1.08 ± 0.51	0.841
≥ 2.0 mg/dL	5/203 (2.5)	1.000	4 (8.0)	0.086	2/105 (1.9)	0.519
Hypokalemia (< 3.5 mmol/L)	52/204 (25.5)	0.155	12/49 (24.5)	0.372	19/104 (18.3)	0.153
C-reactive protein, mg/dL	7.27 ± 5.50	< 0.001	9.82 ± 7.41	< 0.001	1.24 ± 2.64	< 0.001
< 1 mg/dL	7/182 (3.8)	< 0.001	1/49 (2.0)	< 0.001	66/102 (64.7)	< 0.001

*Abbreviations*: *SFTS* severe fever with thrombocytopenia syndrome, *AST* aspartate aminotransferase, *ALT* alanine aminotransferase

^a^*P* value when compared to SFTS

^b^*P* value when compared to SFTS

^c^*P* value when compared to all scrub typhus

^d^Thrombocytopenia, < 80 × 10^3^ /mm^3^

**Table 4:** Major complications during the clinical course (*n* = 362)

Variable	Scrub typhus (*n* = 255)				SFTS	*P* value^c^
	Eschar-positive	*P* value^a^	Eschar-negative	*P* value^b^	*n* = 107	
	*n* = 205		*n* = 50			
CNS involvement						
Altered mentality (GCS < 15)	14/199 (7.0)	< 0.001	8 (16.0)	< 0.001	62/105 (59.0)	< 0.001
Seizure	0/199 (0)	< 0.001	0 (0)	0.002	17/105 (16.2)	< 0.001
Lung involvement						
Mechanical ventilation	5 (2.4)	< 0.001	2 (4.0)	< 0.001	34/106 (32.1)	< 0.001
Renal involvement						
Acute kidney injury	5 (2.4)	< 0.001	5 (10.0)	0.080	23 (21.5)	< 0.001

*Abbreviations*: *SFTS* severe fever with thrombocytopenia syndrome, *CNS* central nervous system, *GCS* Glasgow coma scale

^a^*P* value when compared to SFTS

^b^*P* value when compared to SFTS

^c^*P* value when compared to all scrub typhus

In the multivariate regression analysis, leukopenia (white blood cell count < 4000/mm^3^; odds ratio [OR] 30.13, *P* < 0.001), thrombocytopenia (platelet count < 80,000/mm^3^; OR 19.73, *P* < 0.001) and low CRP (< 1 mg/dL; OR 67.46, *P* < 0.001) were significantly predictive factors for SFTS compared with scrub typhus (Table 5). These 3 factors were consistently significant in a subgroup analysis compared with eschar-negative scrub typhus. As few laboratory results were significantly indicative of scrub typhus in comparison with SFTS in the univariate analysis, primarily only clinical variables were included in the multivariate analysis to identify the predictive factors for scrub typhus. In addition to the characteristic eschar and skin rash, headache was the only consistent risk factor for all cases of scrub typhus and the subgroup of eschar-negative scrub typhus.

**Table 5:** Multivariate analysis for the predictive factors of SFTS compared to those of eschar-positive and negative scrub typhus

Variables	Odds ratio (95% CI)	*P* value
Age	1.03 (0.98–1.09)	0.145
Male gender	2.09 (0.58–7.44)	0.255
Diabetes mellitus	2.53 (0.36–17.43)	0.345
Hypertension	0.64 (0.12–3.28)	0.593
Duration, from onset of illness to initial visit	0.80 (0.68–0.94)	0.008
Altered mentality	2.31 (0.27–19.33)	0.439
Shock	1.72 (0.23–12.68)	0.592
Leukopenia (WBC < 4000/mm^3^)	30.13 (6.08–149.22)	< 0.001
Thrombocytopenia (< 80,000/mm^3^)	19.73 (4.60–84.58)	< 0.001
AST ≥400 IU/L	8.33 (0.39–176.07)	0.173
ALT ≥200 IU/L	0.36 (0.03–4.07)	0.411
LDH ≥800 IU/L	3.05 (0.65–14.15)	0.154
C-reactive protein < 1 mg/dL	67.46 (14.29–318.30)	< 0.001

*Abbreviations*: *SFTS* severe fever with thrombocytopenia syndrome, *CI* confidence interval, *WBC* white blood cell, *AST* aspartate aminotransferase, *ALT* alanine aminotransferase, *LDH* lactate dehydrogenase

A prediction scoring tool for the differential diagnosis of SFTS and scrub typhus was generated using the combination of those 3 parameters (1 point each for WBC count < 4000/mm^3^, platelet count < 80,000/mm^3^ and CRP value < 1 mg/dL); the total score ranged from 0 to 3. On the ROC curve obtained for this model, the optimal cut-off was ≥2. A score ≥ 2 had a sensitivity of 93.1% (95% confidence interval [CI], 87.9–96.4%) and a specificity of 96.1% (95% CI, 93.8–97.6%) for SFTS, with an ROC area under the curve of 0.972 (95% CI, 0.952–0.990) (Table 6). In the eschar-negative scrub typhus subgroup, a score ≥ 2 had a sensitivity of 93.1% (95% CI, 88.9–95.2%) and a specificity of 93.9% (95% CI, 85.0–98.3%) for SFTS. In the subgroup of rash-negative scrub typhus, a score ≥ 2 had a sensitivity of 93.1% (95% CI, 89.0–95.2%) and a specificity of 90.3% (95% CI, 76.8–97.2%) for SFTS (Table 6).

**Table 6:** Diagnostic performance of the SFTS prediction scoring system

SFTS score	Sensitivity (95% CI)	Specificity (95% CI)	Positive likelihood ratio (95% CI)	Negative likelihood ratio (95% CI)
A. SFTS vs. eschar-positive and -negative scrub typhus				
≥2	0.931 (0.879–0.964)	0.961 (0.938–0.976)	23.905 (14.211–39.547)	0.071 (0.037–0.128)
3	0.422 (0.376–0.431)	0.996 (0.976–1.000)	97.382 (15.379–1903.086)	0.581 (0.569–0.640)
B. SFTS vs. eschar-negative scrub typhus				
≥2	0.931 (0.889–0.952)	0.939 (0.850–0.983)	15.212 (5.925–54.850)	0.073 (0.048–0.131)
3	0.422 (0.382–0.422)	1.000 (0.918–1.000)	NA	0.578 (0.578–0.673)
C. SFTS vs. rash-negative scrub typhus				
≥2	0.931 (0.890–0.952)	0.903 (0.768–0.972)	9.624 (3.836–34.539)	0.076 (0.049–0.143)
3	0.422 (0.384–0.422)	1.000 (0.875–1.000)	NA	0.578 (0.578–0.705)

*Abbreviations*: *SFTS* severe fever with thrombocytopenia syndrome, *CI* confidence interval, *NA* not available

## Discussion

A considerable number of SFTS cases occur during the epidemic season of scrub typhus in South Korea. In 2017, 86.7% (9132/10,528) of scrub typhus and 48.1% (131/272) of SFTS cases were officially reported to occur from September to November. Although the case-fatality rate of scrub typhus is low with antibiotic treatment, severe scrub typhus remains an unresolved issue [18, 19]. The concurrent presence of the typical eschar with a compatible clinical manifestation makes the clinical diagnosis of scrub typhus obvious [20], but the poor sensitivity of diagnostic assays for scrub typhus and the presence of eschar-negative scrub typhus make the diagnosis uncertain in some patients. Given this diagnostic uncertainty combined with the potential of severe clinical form, a clinical prediction tool will be very useful to narrow the differential diagnosis for those requiring further urgent investigation [14].

Our prediction tool used 3 variables; leukopenia (WBC count < 4000/mm^3^), thrombocytopenia (platelet count < 80,000/mm^3^) and low CRP (< 1 mg/dL), which could be obtained from routine basic laboratory blood tests and are readily applicable in primary care settings. The cut-off level of thrombocytopenia as platelet count of 80,000/mm^3^ was determined by considering the distribution of platelet counts among the study subjects and the kinetics of initially persistent thrombocytopenia in SFTS [5]. One study previously proposed a similar scoring system using 4 variables: altered mental status, leukopenia, prolonged activated partial thromboplastin time and normal C-reactive protein [21]. In our study, ‘altered mental status’ was not a significant factor to be incorporated into the prediction analysis. We objectively assessed mental status using the Glasgow coma scale, which is one of the basic tools used in critical care. We did not include the coagulation panels in the initial comparison because we do not routinely check coagulation panels during the investigation of possible scrub typhus cases.

A larger sample size might have led to different results. Our study also included a relatively larger number of eschar-negative scrub typhus cases, which causes diagnostic challenges and requires a clinical decision to guide further diagnostic evaluations. We adjusted the comparison with the duration from the onset of illness to the initial presentation because the kinetics of clinical variables are closely time-dependent. The prediction tool worked similarly in all subgroups of scrub typhus (Table 6). The data collection from multiple hospitals in major endemic areas of the two diseases might strengthen the generalizability of our study.

In a binary comparison of clinical parameters, scrub typhus tended to present with more subjective manifestations, whereas SFTS showed more laboratory abnormalities. In a subgroup comparison of eschar-negative scrub typhus with SFTS, skin rash and headache were significantly indicative of scrub typhus. As the skin rash in scrub typhus is a characteristic maculopapular type that is clinically distinct from that of SFTS [20], the presence of a maculopapular skin rash itself has diagnostic value if the clinician is sufficiently experienced. However, approximately 10% of patients with scrub typhus are reported to lack the typical skin rash [22, 23]. Therefore, scrub typhus without eschar and a skin rash poses a further diagnostic challenge. In our study, 12.2% of eschar-positive and 22.0% of eschar-negative scrub typhus patients had no skin rash. The rate of eschar-negative scrub typhus has been reported to be approximately 10%, although a skilled physician may observe a different rate [22, 23].

Reports regarding co-infection of both scrub typhus and SFTS are limited. In a recent report, 23.0% of patients clinically suspected of scrub typhus were SFTS-positive [24]. There was a case report of co-infection diagnosed by PCR [25]. In another report, however, none of the 38 patients with scrub typhus were SFTS-positive, whereas one of 21 patients with SFTS was serologically suggestive of scrub typhus. Thus, the clinical evidence on the possibility of co-infection is not solid, and further monitoring is necessary. From an ecological perspective, given the vectors of the two diseases, co-infection is not likely. Although the epidemic seasons overlap and there is a risk of simultaneously acquiring the two diseases during outdoor activity, they do not share vectors, and the ecologies of their vectors differs. Phenotypically, scrub typhus is highly prevalent in the rice field areas of western and southwestern South Korea [26], whereas the incidence of SFTS is low in this area, consistent with the low SFTSV infection rate in ticks. Conversely, the incidence of SFTS is high in the eastern and southeastern mountainous area of South Korea [5, 27].

The causative agent of scrub typhus, *O. tsutsugamushi*, is transmitted by trombiculid chigger mites. The causative *Trombiculidae* have a nationwide distribution in South Korea [28, 29]. Although the larvae of trombiculid mites can parasitize most animals, rodents and some other small mammals are their primary hosts [30, 31]. The prevalence and abundance of chigger mites on small mammals are much higher in cultivated flatland landscapes [32]. Meanwhile, *Haemaphysalis longicornis* is the predominant vector for SFTSV, but other tick species such as *H. flava*, *Amblyomma testudinarium* and *Ixodes nipponensis* can also carry SFTSV in South Korea [27, 33, 34]. *H. longicornis* is able to transmit SFTSV via both transovarial and transstadial modes [35]. *H. longicornis* is widely distributed in Australia, New Zealand, Korea, Japan and China [36]. Larger mammals, such as rabbits, badgers, deer and wild boars, rather than rodents, have been suggested to be the principal hosts for this tick species [36]*.* The differences between the main hosts and habitats of the vectors makes their simultaneous exposure less likely. However, the enhanced intrusion of wild animals into cultivated farmland areas and serologic evidence of SFTSV infection in domestic animals may indicate the exposure risk to dual vectors [37, 38]. The case-fatality trend of scrub typhus also deserves to be mentioned for indirect evidence of co-infection. Although there are approximately 10,000 cases of scrub typhus annually in South Korea, the recent annual mortality was stable in the range of 11–13 cases (http://www.cdc.go.kr/npt). This suggests that co-infection is rare, considering the high mortality and rising incidence of SFTS.

The magnitude of the prevalence of SFTS and the need for intense differential vigilance in the community must be further investigated. The seroprevalence for SFTSV antibodies in South Korea were reported to be 2.7 to 7.7% in rural area and 1.9% in urban areas in small-scale studies [39, 40]. SFTS is already endemic throughout South Korea, and the rapidly increasing trend of its incidence is obvious [5]. The severity and poor prognosis of SFTS demand accurate initial clinical triage. Human granulocytic anaplasmosis and human monocytotrophic ehrlichiosis which have similar clinical presentations are also important differential diseases. Hence, we suggest a clinical decision algorithm based on our findings as follows. In an atypical febrile disease during the epidemic season of scrub typhus, the empirical administration of doxycycline is desirable. Positive findings of eschar or a maculopapular skin rash on physical examination are strongly suggestive of scrub typhus and are an indication for maintaining doxycycline treatment. If there is no eschar or maculopapular rash, the calculation of the prediction score in our study may guide the degree of suspicion for SFTS. Until confirming the presence of SFTS, continuing doxycycline may be clinically useful to cover eschar- and skin rash-negative scrub typhus, anaplasmosis, and ehrlichiosis, which all respond to doxycycline.

This study has several limitations. First, the prediction scoring tool was evaluated only in comparison with scrub typhus. Further performance assessment in general febrile patients may be useful to demonstrate the utility of this tool for clinically identifying SFTS. Second, although the SFTS data were primarily obtained from intensive clinical care settings, the retrospective nature of data collection might lead to a bias in contrast to the prospective collection of scrub typhus cases. In addition, only viremic SFTS cases were included, which might exclude mild cases. However, our concentration on moderate to severe cases might have greater clinical impacts in practice. Third, we did not evaluate other parameters such as activated partial thromboplastin time or ferritin, which have also been suggested as useful markers for SFTS, because the previous scrub typhus studies did not include those variables [21, 41]. However, these laboratory variables are not readily available at a point of care or are not necessary for usual clinical practices in the primary care settings where the confirmatory assays for both diseases are not available. Lastly, we used several cohorts of different time points. As there is no evidence that the clinical features of scrub typhus and SFTS have changed respectively, and we have used a same methodology, mixing of cohorts might not lead to the significant inhomogeneity.

## Conclusions

We suggested a clinical prediction scoring tool for SFTS in comparison with scrub typhus that consists of 3 variables: leukopenia (WBC count < 4000/mm^3^), thrombocytopenia (platelet count < 80,000/mm^3^) and low CRP (< 1 mg/dL). It is a simple and readily applicable tool that can be used in primary care settings. It will be useful for differentiating between SFTS and eschar- and skin rash-negative scrub typhus. We also showed an in-depth comparison of SFTS and scrub typhus to better understand the clinical features of both diseases. This tool may also be used to screen out SFTS in areas where SFTS has not yet been reported but is geographically capable of existing because of proximity to the endemic countries.

## Abbreviations

ALT: Alanine transaminase
AST: Aspartate transaminase
CI: Confidence interval
CRP: C-reactive protein
IFA: Indirect immunofluorescence antibody assay
KCDC: Korea Centers for Disease Control and Prevention
LDH: Lactate dehydrogenase
OR: Odds ratio
PCR: Polymerase chain reaction
RT: Reverse transcription
SFTS: Severe fever with thrombocytopenia syndrome
SFTSV: SFTS virus
